# The PSEN1 E280G mutation leads to increased amyloid-β43 production in induced pluripotent stem cell neurons and deposition in brain tissue

**DOI:** 10.1093/braincomms/fcac321

**Published:** 2022-12-07

**Authors:** Nanet Willumsen, Charles Arber, Christopher Lovejoy, Jamie Toombs, Argyro Alatza, Philip S J Weston, Lucia Chávez-Gutiérrez, John Hardy, Henrik Zetterberg, Nick C Fox, Natalie S Ryan, Tammaryn Lashley, Selina Wray

**Affiliations:** Department of Neurodegenerative Disease, UCL Queen Square Institute of Neurology, London WC1N 1PJ, UK; The Queen Square Brain Bank for Neurological Disorders, Department of Clinical and Movement Neuroscience, UCL Queen Square Institute of Neurology, London WC1N 1PJ, UK; Department of Neurodegenerative Disease, UCL Queen Square Institute of Neurology, London WC1N 1PJ, UK; Department of Neurodegenerative Disease, UCL Queen Square Institute of Neurology, London WC1N 1PJ, UK; Department of Neurodegenerative Disease, UCL Queen Square Institute of Neurology, London WC1N 1PJ, UK; UK Dementia Research Institute, University College London, London WC1E 6AU, UK; Department of Neurodegenerative Disease, UCL Queen Square Institute of Neurology, London WC1N 1PJ, UK; Department of Neurodegenerative Disease, UCL Queen Square Institute of Neurology, London WC1N 1PJ, UK; Dementia Research Centre, UCL Queen Square Institute of Neurology, London WC1E 6BT, UK; VIB Center for Brain and Disease Research, 3000 Leuven, Belgium; Department of Neurology, KU Leuven, 3000 Leuven, Belgium; Department of Neurodegenerative Disease, UCL Queen Square Institute of Neurology, London WC1N 1PJ, UK; UK Dementia Research Institute, University College London, London WC1E 6AU, UK; Department of Neurodegenerative Disease, UCL Queen Square Institute of Neurology, London WC1N 1PJ, UK; UK Dementia Research Institute, University College London, London WC1E 6AU, UK; Department of Psychiatry and Neurochemistry, Institute of Neuroscience and Physiology, Sahlgrenska Academy at University of Gothenburg, S-431 80 Mölndal, Sweden; Department of Neurodegenerative Disease, UCL Queen Square Institute of Neurology, London WC1N 1PJ, UK; Dementia Research Centre, UCL Queen Square Institute of Neurology, London WC1E 6BT, UK; UK Dementia Research Institute, University College London, London WC1E 6AU, UK; Department of Neurodegenerative Disease, UCL Queen Square Institute of Neurology, London WC1N 1PJ, UK; Dementia Research Centre, UCL Queen Square Institute of Neurology, London WC1E 6BT, UK; UK Dementia Research Institute, University College London, London WC1E 6AU, UK; Department of Neurodegenerative Disease, UCL Queen Square Institute of Neurology, London WC1N 1PJ, UK; The Queen Square Brain Bank for Neurological Disorders, Department of Clinical and Movement Neuroscience, UCL Queen Square Institute of Neurology, London WC1N 1PJ, UK; Department of Neurodegenerative Disease, UCL Queen Square Institute of Neurology, London WC1N 1PJ, UK

**Keywords:** induced pluripotent stem cells, Alzheimer’s disease, γ-secretase, APP, amyloid-β

## Abstract

Mutations in the presenilin 1 gene, *PSEN1,* which cause familial Alzheimer’s disease alter the processing of amyloid precursor protein, leading to the generation of various amyloid-β peptide species. These species differ in their potential for aggregation. Mutation-specific amyloid-β peptide profiles may thereby influence pathogenicity and clinical heterogeneity. There is particular interest in comparing mutations with typical and atypical clinical presentations, such as E280G. We generated *PSEN1* E280G mutation induced pluripotent stem cells from two patients and differentiated them into cortical neurons, along with previously reported *PSEN1* M146I, *PSEN1* R278I and two control lines. We assessed both the amyloid-β peptide profiles and presenilin 1 protein maturity. We also compared amyloid-β peptide profiles in human post-mortem brain tissue from cases with matched mutations. Amyloid-β ratios significantly differed compared with controls and between different patients, implicating mutation-specific alterations in amyloid-β ratios. Amyloid-β42:40 was increased in the M146I and both E280G lines compared with controls. Amyloid-β42:40 was not increased in the R278I line compared with controls. The amyloid-β43:40 ratio was increased in R278I and both E280G lines compared with controls, but not in M146I cells. Distinct amyloid-β peptide patterns were also observed in human brain tissue from individuals with these mutations, showing some similar patterns to cell line observations. Reduced presenilin 1 maturation was observed in neurons with the *PSEN1* R278I and E280G mutations, but not the M146I mutation. These results suggest that mutation location can differentially alter the presenilin 1 protein and affect its autoendoproteolysis and processivity, contributing to the pathological phenotype. Investigating differences in underlying molecular mechanisms of familial Alzheimer’s disease may inform our understanding of clinical heterogeneity.

## Teaser

Willumsen *et al*., generate patient-derived induced pluripotent stem cells containing the Presenilin 1 E280G mutation. The E280G lines displayed a high relative production of Abeta43, a finding matched in post-mortem tissue with the same mutation. This mutation also disrupts presenilin 1 protein maturity. Together these findings indicate mutation-specific pathogenic mechanisms.

## Graphical Abstract

**Graphical abstract:**
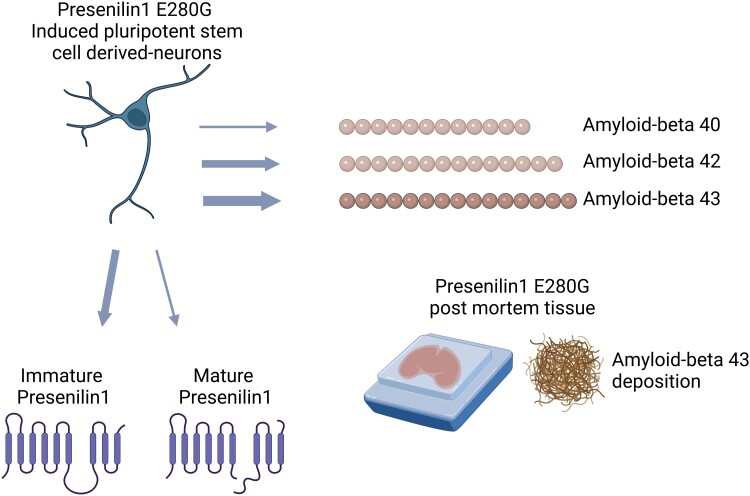

## Introduction

Alzheimer’s disease is defined by the cerebral accumulation of pathological misfolded proteins into amyloid beta (Aβ) plaques and neurofibrillary tangles containing tau.^1–3^ Familial Alzheimer’s disease (FAD) shares these pathological features with sporadic Alzheimer’s disease (sAD). However, FAD is caused by rare autosomal dominant mutations in the presenilin 1 (*PSEN1), PSEN2* or amyloid precursor protein (*APP)* genes and *APP* duplications.^4–9^

The presenilin 1 protein (PSEN1) is the catalytic component of the γ-secretase complex, responsible for the cleavage of APP.^10,11^ In the amyloidogenic pathway, APP is cleaved by β-secretase (BACE1) releasing the sAPPβ fragment.^12^ The remaining membrane-bound C99 fragment undergoes an initial epsilon cleavage, releasing an APP intracellular C domain and a membrane-bound Aβ peptide. Sequential carboxypeptidase-like cleavages of Aβ by the γ-secretase complex releases Aβ peptides of varying length.^13^ Epsilon Aβ cleavage occurs at residue 48 or 49, giving rise to two cleavage pathways of decreasing peptide length (Aβ49 > 46 > 43 > 40 and Aβ48 > 45 > 42 > 38).^14^ In *PSEN1*-FAD, there is a loss of processivity leading to qualitative shifts towards longer Aβ peptides.^15^

Recently, there have been multiple papers that have generated induced pluripotent stem cells (iPSCs) from FAD and Down’s syndrome patient fibroblasts, reviewed in.^16^ Studies have revealed that specific FAD mutations produce very distinct Aβ ratios, as shown in both iPSC differentiated into neurons^17^ and in other cell-based and activity assay models,^18–20^ recapitulating aspects of disease *in vitro*. Importantly, these Aβ ratios are likely to reflect early perturbations in Aβ processing, revealing early disease mechanisms.

Compared with wild-type (WT) *PSEN1*, most pathogenic *PSEN1* mutations cause an increase in the ratio of Aβ42 to total Aβ, and notably an increased Aβ42:40 ratio.^21^ However, the mechanism by which ratios are altered differs by mutation. Specific *PSEN1* mutations affecting γ-secretase activity can influence both absolute amounts of Aβ and the Aβ cleavage pathway selection, leading to altered ratios of the peptides within those pathways.^22^

Pathogenic *PSEN1* mutations appear to consistently hinder sequential carboxypeptidase cleavage, leading to the release of longer Aβ peptides, alterations of which can be measured via the Aβ42:38 and Aβ43:40 ratios. This results in a relative increase in, and release of, longer Aβ peptides which are more prone to aggregation,^6,15,22^ in turn leading to more oligomeric Aβ and greater extracellular deposition.^23^ Mutations that severely compromise γ-secretase carboxypeptidase activity affect residues that map near the proposed γ-secretase active site.^20,24^

Upon γ-secretase complex assembly full-length PSEN1 undergoes autoendoproteolysis to produce cleaved PSEN1, consisting of the PSEN1 N-terminal fragment (NTF) and C-terminal fragment.^25^ Mutations near the endoproteolytic site are proposed to inhibit endoproteolysis,^26^ which can lead to altered processivity.^27^ Interestingly, even within the γ-secretase complex bearing full-length PSEN1, the catalytic pore is still able to be formed,^28^ which suggests that proteolytic activity can still occur, although it may be reduced.

In some mutations, an increase in the ratio of Aβ43 compared with other peptide lengths or increased absolute levels has been linked to an increase in the full-length form of PSEN1. The *PSEN1* R278I mutation has previously been shown to have increased full-length PSEN1 and Aβ43 ratio in patient iPSC-derived neuronal lines by our group.^17^ Increased Aβ43 and full-length PSEN1, with reduced Aβ40, was also observed in the R278I mutation mouse embryonic fibroblast CF-1 (MEF) cell line^19,29,30^ and mouse models.^30^ Importantly, alterations in PSEN1 maturity were not caused by differences in *PSEN1* expression, and *APP* expression was not altered,^17^ implying specific mutations affect disease via influencing PSEN1 structure/function. Along with R278I, other mutations associated with high Aβ43 are located around the intracellular loop. Another important mutation located in this area is the E280G mutation. Clinically, the E280G mutation is associated with atypical clinical presentations, including spastic paraparesis and marked white matter hyperintensities on MRI.^31,32^ Pathologically, a brain biopsy from a *PSEN1* E280G carrier showed numerous large non-cored Aβ deposits as cotton wool plaques (CWPs) and severe cerebral amyloid angiopathy (CAA).^31^ An individual with the *PSEN1* E280Q mutation has also had a similar CWP pathology observed post-mortem.^33^ HEK lines expressing the E280G mutation show significantly increased Aβ42 concentration, non-significantly increased Aβ40 concentration and an overall significantly increased Aβ42:40 ratio in conditioned media compared with controls.^34^ However, as discussed by the authors, these results suffer from the caveat that only mutated *PSEN1* is expressed in these lines compared with heterozygous mutation carriers where WT PSEN1 is also expressed. In another study, cloned γ-secretase with a range of PSEN1 mutations was purified, WT or mutant γ-secretase was mixed with APP-C99 in a detergent-based assay and incubated at 37°C for 16 h. Aβ peptides produced in the detergent-based assay were measured using an AlphaLISA assay. The assays revealed greater amounts of Aβ42 compared with Aβ40 and a higher Aβ42:40 ratio in association with the *PSEN1* E280G mutation, while western blot images also suggested the presence of full-length PSEN1, possibly due to reduced endoproteolytic processing.^20^ Aβ43 has not, however, been assessed in the *PSEN1* E280G mutation.

In this study, we generated E280G iPSCs from two clinically affected FAD patient donors. We went on to investigate Aβ profiles using ratios measured via an enzyme-linked immune-sorbent assay (ELISA) and an electrochemiluminescence assay, and PSEN1 protein maturation in iPSC-derived neurons, with the hypothesis that the E280G mutation decreases PSEN1 autoendoproteolysis, resulting in the enhanced generation of Aβ43, similar to the proximal *PSEN1* R278I mutation. Finally, we compared these findings to post-mortem tissue from E280G carriers.

## Materials and methods

### Cell culture

Cell lines used in this study are summarized in Table 1. The study was approved by the joint research ethics committee of the National Hospital for Neurology and Neurosurgery and the Institute of Neurology (09/H0716/64), and informed consent was gained for all samples. All reagents were purchased from Thermo Fisher Scientific unless specified. Fibroblasts were cultured as previously described.^35^ Fibroblasts underwent episomal reprogramming according to Okita *et al*.^36^ Fibroblasts underwent nucleofection with episomal DNA coding for OCT4 and shRNA against p53, KLF4, SOX2 and c-MYC using plasmids #27077, #27078, and #27080 which were obtained from Addgene. Nucleofection was performed using the Lonza P2 Nucleofection kit (Amaxa PBP2-00675) as per manufacturer’s instructions.

**Table 1:** Cell lines used in this study

Line name	Mutation	Sex	age at onset	Age at biopsy	APOE genotype	Origin
CTL1 (SIGi001-A-1)	—	F	—	24	3/4	Sigma Aldrich/EBiSC
CTL2 (RBi001-A)	—	M	—	45–49	3/3	Sigma Aldrich/EBiSC
M146I	*PSEN1* M146I	M	Presymptomatic	38	3/3	StemBancc
R278I	*PSEN1* R278I	M	58	60	2/4	Arber et al. 2019
E280G.A	*PSEN1* E280G	M	41	45	3/3	Generated in this study
E280G.B	*PSEN1* E280G	M	38	49	3/4	Generated in this study

After nucleofection, cells were grown on geltrex (1:100, DMEM/F12) (A1413302, Life Technologies), maintained in fibroblast media which was changed every three days.

Six days after electroporation, cells were lifted with 1 × trypsin (15090-046, Gibco) and resuspended in fibroblast media on cultured on geltrex-coated MEF feeder layer coated (ATCC® SCRC-1040^TM^) 10 cm dishes (172958, Nunc). On Day 8 after nucleofection, media was switched to human embryonic stem cell media (DMEM/F12 Glutamax, 20% Knock out Serum, 2 mM L-glutamine, 1 × Non-essential amino acids, 50 U/ml pen & 50 µg/ml strep, all from Gibco, 50 µM 2-Mercaptoethanol (ThermoFisher), 20 ng/ml Fibroblast Growth Factor-basic (Peprotech)) and changed daily. Nascent iPSC colonies were picked and transferred into feeder-free conditions (Essential eight media and geltrex matrix). iPSCs were passaged using 0.5 mM ethylenediaminetetraacetic acid.

iPSCs were differentiated into cortical neurons according to the Shi *et al.*^37^ protocol. Briefly, iPSCs were grown to 100% confluence, and the media was changed to neural induction media [N2B27 containing 10 μM SB431542 (Tocris) and 1 μM dorsomorphin (Tocris)]. N2B27 media consists of a 1:1 mixture of Dulbecco’s modified eagle medium F12 (DMEM-F12) and Neurobasal supplemented with 0.5X N2, 0.5X B27, 0.5X non-essential amino acids, 1 mM L-glutamine, 25 U/ml pen & 25 µg/ml strep, 50 μM β-mercaptoethanol and 2.5 μg/ml insulin. At Day 12, the precursors were passaged by using a cell scraper (541070, Greiner Bio-One International) onto laminin-coated wells and at Day 18 passaged using dispase and plated in laminin-coated wells (Sigma) in N2B27 media. The final passage was performed at Day 35 using accutase and maintained in N2B27 media on poly-L-ornithine (Sigma) and laminin (Sigma) coated wells in N2B27 media until the required time point. Neurons were analysed at 100 days post-neural induction. One hundred days were selected as it represents the end of corticogenesis and detectable levels of secreted Aβ.^38^

### Karyotyping

DNA was harvested using the TRIzol™ (15596026, Invitrogen) protocol according to manufacturer’s instructions. Briefly, cells were homogenized in 1 ml TRIzol™ followed by the addition of 200 µl Chloroform (22711.290, VWR), shaken vigorously and then incubated for 3 mins followed by centrifugation at 12 000g*_(av)_,* 4^°^C for 15 mins. After isolating and removing the aqueous phase, the DNA-containing interphase was removed from the organic phase by adding 300 µl of 100% ethanol was added and samples were shaken, incubated for 3 mins and centrifuged at 2000 g*_(av)_,* 4^°^C for 5 mins. The DNA was washed twice by the addition of 0.1 M trisodium citrate in 10% ethanol, pH 8.5 followed by incubation for 30 mins with regular inversion followed by centrifugation at 2000g*_(av)_,* 4^°^C for 5 mins. The pellet was resuspended in 1.5 ml 75% ethanol and incubated for 20 mins with regular inversion followed by centrifugation at 2000 g*_(av)_,* 4°C for 5 mins. The pellet was air dried for 10 mins before resuspension in 300 µL 8 mM NAOH, followed by centrifugation at 12 000g*_(av)_,* 4^°^C for 10 mins. Genomic DNA from the two patient *PSEN1* E280G lines, named E280G.A and E280G.B, were analysed for chromosomal abnormalities using the hPSC Genetic Analysis Kit (Stem Cell Technologies) and the StemCell genetic analysis app. Amplification in a minimal critical region of chromosome 17 of the E280G.A line was observed, with a calculated copy number of 2.44. There were no significant amplifications in the E280G.B line. Additional chromosomal sites (Chr 1q, Chr 4p, Chr 8q, Chr 10p, Chr 12p, Chr 18q and Chr Xp) showed no significant abnormalities.

### NanoString Stem Cell Characterization Panel

RNA was harvested from iPSCs 2 days after passage via Monarch total RNA miniprep kit (NEB). 100 ng was used for Nanostring analysis on the nCounter Stem Cell Characterization Panel. The expression of 770 genes was analysed on the nSolver software to compare grouped control (CTL1 and CTL2) and grouped E280G (E280G.A and E280G.B) data. Linear regression analysis was performed using GraphPad Prism.

### Immunocytochemistry

Coverslips were washed with Dulbecco’s phosphate-buffered saline (PBS) and fixed for 15–20 min in 4% formaldehyde followed by excess 1X PBS wash and stored in PBS at 4^°^C. Coverslips were blocked in 5% BSA/0.3% PBS-triton-X-100 followed by primary antibody incubation (Table 2) in block solution for 2 hrs or 4^°^C overnight. After three washes in 1X PBS-0.3% triton, a secondary antibody (Table 2) in block solution was added for 1 h, in the dark. Following a PBS-0.3% triton wash, DAPI (1:5000, D1306, Invitrogen) was added as a nuclear counterstain and incubated in the dark. Cells were washed once with PBS-triton followed by 1X PBS, mounted onto slides with fluorescence mounting medium (S3023, DAKO) and imaged on a Leica DM5500 B microscope using Leica Application suite X software (Leica microsystems, Wetzlar, Germany).

**Table 2:** Antibodies used in this study

Antibody	Company	Species	Dilution	Purpose
SSEA4	Biolegend MC-813–70	Mouse	1:300	ICC
OCT4	Santa Cruz SC-8628	Rabbit	1:400	ICC
NANOG	Cell Signaling Technology D73G4	Rabbit	1:500	ICC
FOXG1	Abcam Ab18259	Rabbit	1:500	ICC
PAX6	DSHB	Mouse	1:5	ICC
TBR1	Abcam AB31940	Rabbit	1:300	ICC
TUJ1	Biolegend 801201	Mouse	1:1000	ICC
TUJ1	Biolegend 802001	Rabbit	1:1000	ICC
488 Donkey anti-Rabbit	Alexa Fluor® A21206		1:5000	ICC
594 Donkey anti-Mouse	Alexa Fluor® A21203		1:5000	ICC
488 Donkey anti-mouse	Alexa Fluor® A-21202		1:5000	ICC
594 Donkey anti-Rabbit	Alexa Fluor® A21207		1:5000	ICC
PSEN1 N-term	Millipore MAB1563	Rat	1:500	Western blot
β-Actin	Sigma	Mouse	1:1000	Western blot
Goat anti-rat	Alexa Fluor® A21096		1:10,000	Western blot
Goat anti-mouse	Li-cor 926-68071		1:10 000	Western blot
Aβ40	Merk AB5074P	Rabbit	1:100	IHC
Aβ42	BioLegend 805509	Mouse	1:500	IHC
Aβ43	BioLegend 805607	Mouse	1:500	IHC
Swine anti-rabbit	DAKO E0353		1:200	IHC
Rabbit anti-mouse	DAKO E0354		1:200	IHC

### Conditioned cell media collection

Cortical neurons were incubated in N2B27 media for 48 h prior to collection, as described previously.^17^ Conditioned media was centrifuged at 2000 g_(av)_ for 5 min at RT, with 1 ml aliquoted in Sarstedt 2 mL PP tubes (72.694.406) and stored at −80^°^c.

### Western blotting

Cells that had been used for conditioned media collection were lysed in radioimmunoprecipitation assay buffer (RIPA) containing phosphatase and protease inhibitors (Roche). Using the Bio-Rad *DC*^TM^ Protein Assay, 5 µl of sample and standards (1:1 dilutions of Bovine Serum Albumin in RIPA from 4 mg/ml to 0.125 mg/ml + a RIPA blank) was added to reagent A + S, followed by reagent B, in triplicate. After plate shaking and 15 min development time, absorbance was measured at 750 nm on a Spark^TM^ 10 m plate reader (Tecan). Concentration was calculated from the standard curve using the equation x = (y-m)/b. Lysates of 30 µg loaded with NuPage^TM^ LDS sample buffer and NuPAGE^TM^ reducing agent were denatured at 70^°^c for 10 min followed by brief centrifugation. Electrophoresis was conducted on a NuPage^TM^ 10% Bis-Tris gel in MES SDS running buffer at 150 V for 1 h 30 mins. Transfer to nitrocellulose membrane was conducted at 30 V for 1 h. The membrane was blocked in 5% BSA/0.1% PBS-tween20 (PBS-T) and incubated in anti-PSEN1 overnight at 4°C. The membrane was washed three times with 0.1% PBS-T and incubated in a secondary antibody (Table 2) for 1 h, in the dark. The membrane was washed once with 0.1% PBS-T and twice with PBS before imaging on an Odyssey Fc (Li-cor). The membrane was then incubated for 1 h in anti-β-actin, washed three times with 0.1% PBS-T and incubated in a secondary antibody (Table 2) for 1 h, in the dark. The membrane was washed once with 0.1% PBS-T and twice with PBS before imaging on an Odyssey Fc (Li-cor). Data from the two control lines (CTL1 and CTL2) were pooled and comparisons were made between all cell lines.

### Immunoassays

Solid phase sandwich, ELISA for Aβ1-43 (27710, IBL) was conducted using D100 neuron-conditioned cell media according to manufacturer’s instructions. Briefly, 100 µl of calibrator, control and samples were added in duplicate to individual wells coated with an Aβ1-43 peptide-specific capture antibody. A labelled detection antibody (82E1) was applied followed by chromogen incubation and the eventual addition of the stop solution. The plate was then read at 450 nm, with a reference of 650 nm (FLUOstar Omega Microplate Reader, BMG Labtech). Aβ38/40/42 in D100 neuron-conditioned cell media were measured by electrochemiluminescence using the V-PLEX Aβ Peptide Panel 1 (6E10) Kit (1 Plate) (K15200E, Mesoscale Discovery), according to manufacturer’s instructions. Briefly, samples were diluted 1:1 in diluent 35 and added in duplicate to individual wells which were coated in mouse monoclonal peptide-specific capture antibodies for human Aβx-38/x-40/x-42. Samples were incubated with anti-Aβ antibody (6E10 clone) as the detection antibody conjugated with an electrically excitable SULFO-TAG. Measurements were made using the MESO QuickPlex SQ 120, MSD. Concentrations were calculated from the ECL signal using a four-parameter logistic curve-fitting method with the MSD Workbench software package. Absolute levels of secreted Aβ43, Aβ42, Aβ40, and Aβ38 were measured and converted to ratios for comparison. Data from the two control lines (CTL1 and CTL2) were pooled and comparisons were made between all cell lines, except for the E280G.B line in Aβ38 analyses due to the low number of inductions (*n* = 2).

### Complementary DNA synthesis and quantitative PCR (qPCR)

RNA was harvested using the TRIzol™ (15596026, Invitrogen) protocol and stored at −80^°^c. Complementary DNA was generated from 500 ng of harvested RNA using the SuperScript IV Reverse Transcription Kit (18091050, ThermoFisher) and random hexamers following the manufacturer’s instructions. qPCR for each induction (run in triplicate) was performed using Power SYBR™ Green (Thermo Fisher) on the Mx3000P qPCR system (Aligent). The following primers were used: T-box Brain Transcription Factor 1 (TBR1, F: AGCAGCAAGATCAAAAGTGAGC R: ATCCACAGACCCCCTCACTAG), Special AT-rich sequence-binding protein 2 (SATB2, F: CAACGCAACTAATAATCATCTCCC R: GAGAAAGGGCTGAGAACCCG), Class III beta-tubulin (TUJ1, F: CATGGACAGTGTCCGCTCAG R: CAGGCAGTCGCAGTTTTCAC) and PSEN1 (F: CAATACTGTACGTAGCCAGA R: AATGGGGTATAGATTAGCTG). Data were normalized to housekeeping gene *RPL18a* (F: CCCACAACATGTACCGGGAA R: TCTTGGAGTCGTGGAACTGC).

### Immunohistochemistry

All three FAD cases assessed in this study were obtained through the brain donation programme of the Queen Square Brain Bank for Neurological Disorders, Department of Clinical and Movement Neurosciences, UCL Queen Square Institute of Neurology. The protocols used for brain donation and ethical approval for this study were approved by a London Research Ethics Committee and tissue is stored for research under a license from the Human Tissue Authority. The standard diagnostic criteria for the neuropathological diagnosis of Alzheimer’s disease were used in all cases.^1–3,39^ For a summary of cases, see Table 3. Paraffin-embedded serial sections of 8 μm thickness from the temporal cortex were cut. Serial sections were used for Aβ immunohistochemistry (IHC). Slides were pre-treated in formic acid followed by a pressure cooker in citrate buffer pH 6.0. Endogenous peroxidase activity was blocked with 0.3% H_2_0_2_ in methanol and non-specific binding with 10% dried milk solution. Sections were incubated with the primary antibodies (Table 2) overnight at 40°C, followed by biotinylated secondary antibody ABC complex (30 minutes; PK-6100, Vector Laboratories Ltd). Colour was developed with di-aminobenzidine/H_2_0_2._ Micrographs were taken on a brightfield microscope and images analysed for percentage area staining using Halo software (Indica Labs) with the Indica Labs standard macro (Area Quantification v1) optimized for each Aβ antibody.

**Table 3:** Summary of post-mortem brain tissue used in this study

Mutation	*APOE* genotype	Sex	Age at onset	Disease duration (years)	Braak Tau	Thal Phase	CERAD	PM delay
								(hrs/mins)
*PSEN1* M146I	23	M	48	6	>6	5	Frequent	115:35:00
*PSEN1* R278I	23	M	54	12	6	5	Frequent	77:45:00
*PSEN1* E280G	34	F	42	11	6	5	Frequent	11:00

### Statistical analysis

Cell data were checked for normality using QQ plots. For statistical analysis, the CTL1 and CTL2 lines have been pooled. When needed for western blot and qPCR analysis, CTL2 was used for normalization. Cell data were assessed using one-way ANOVA with a probability value of less than *P* < 0.05 considered significant. When ANOVA analysis reached statistical significance, Tukey’s *post hoc* test was used, adjusting the significant level appropriately for the analysis conducted. All cell data statistics were conducted on GraphPad Prism.

## Results

### Generation and characterization of iPSCs from two *PSEN1* E280G mutation carriers

**Figure 1:**
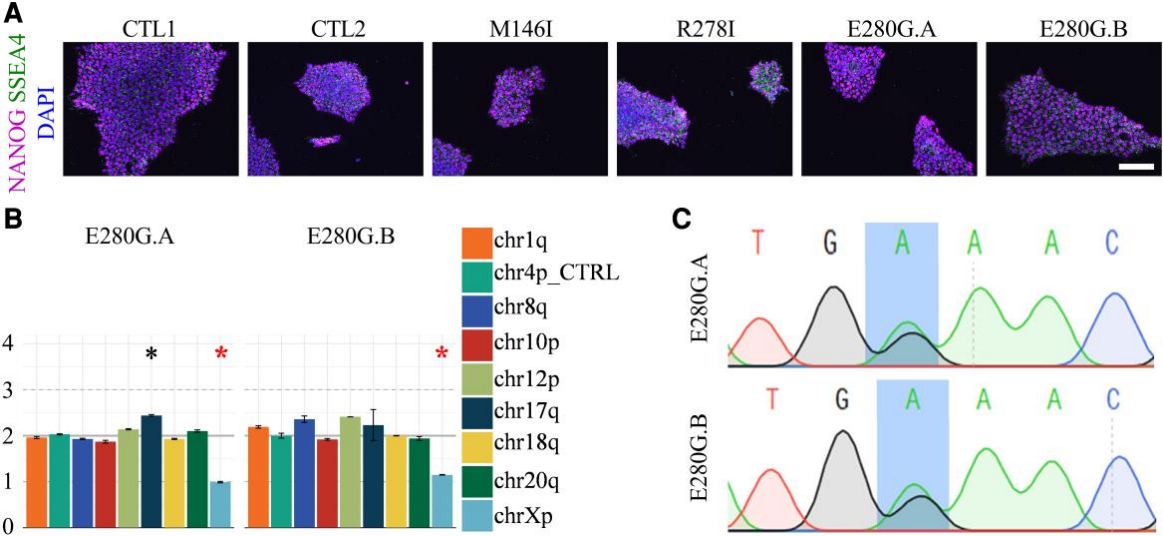
**iPSC generation and characterization of new E280G lines.** (**A**) Representative images of iPSC lines show expression of endogenous pluripotency markers in all lines. NANOG, magenta; SSEA4, green; DAPI, blue. Scale bar represents 100 µm. (**B**) Karyotype stability analysis via genomic PCR. Compared with controls, a significant amplification indicated (green asterisk *) for primer pair chr17q in the E280G.A line and the red asterisk indicates significant difference for chrXp primer pair, confirming male sex of samples. (**C**) Sequencing reads in *PSEN1* exon 8 confirm E280G SNP (A > G) in one allele, confirming heterozygous mutation in both cell lines. Red box outlines E280G SNP location.

Fibroblasts from two *PSEN1* E280G mutation carriers were reprogrammed to iPSCs via episomal reprogramming. Confirmation of successful reprogramming in the newly generated *PSEN1* E280G lines was confirmed by immunocytochemistry (ICC) which showed that all iPSC lines expressed the markers of pluripotency NANOG and SSEA4 (Fig. 1A). Chromosomal analysis indicated stable karyotypes with the exception of a potential amplification in a minimal critical region of chromosome 17 in the *PSEN1* E280G.A line, with a calculated average copy number of 2.44 (Fig. 1B). The exact primer probe binding sequence is not disclosed by the StemCell technologies thus the exact region of amplification cannot be confirmed. Amplifications in 17q are commonly found in hPSC cultures indicating a pro-survival benefit to stem cells.^40^ Expression of 770 stem cell-associated genes was analysed on the NanoString Stem Cell Characterization Panel. Analysis of grouped data to compare control lines (CTL1 and CTL2) with E280G lines (E280G.A and E280G.B) displayed a high correlation (R^2^ = 0.9932), representing a similar stem cell expression profile in the newly derived lines compared with established iPSCs (Supplementary Fig. 1). Sanger sequencing of genomic DNA obtained from iPSCs confirmed the presence of the E280G mutation within both cell lines (Fig. 1C).

### Differentiation of E280G iPSC into cortical neurons

**Figure 2:**
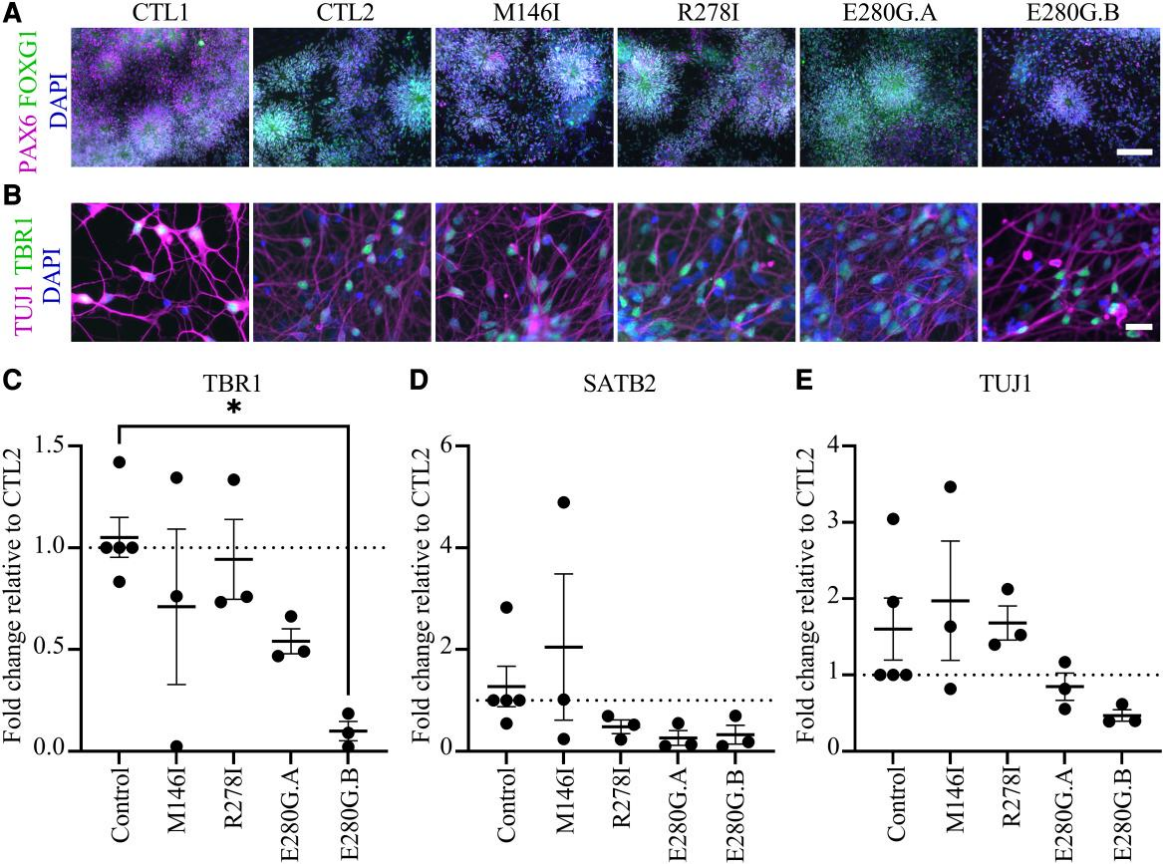
**Characterization of iPSC-cortical neurons.** Control, M146I, R278I and E280G iPSC lines were differentiated into cortical neurons. (**A**) Confirmation of cortical neurogenesis in D25 cortical neurons. Representative images shown. DAPI, blue; PAX6, magenta; FOXG1, green. White scale bar represents 100 µm. (**B**) Confirmation of neuronal phenotype in D50 cortical neurons. DAPI, blue; neuronal tubulin (TUJ1), magenta; deep layer cortical neuronal marker TBR1, green. White scale bar represents 20 µm. (**C**) TBR1 expression measured by qPCR significantly differed across cell lines. (**D**) SATB2 expression measured by qPCR did not significantly differ across cell lines. (**E**) TUJ1 expression measured by qPCR did not significantly differ across cell lines. *n* = 3 inductions per cell line with each individual dot representing qPCR data from triplicate technical repeats, except in the CTL1 line where *n* = 2 inductions. Error bars represent mean and SEM. One-way ANOVA with Tukey’s multiple comparison, *P < 0.05

Together with two control lines, and two previously characterized FAD iPSC lines (*PSEN1* M146I and R278I),^17^ the newly generated lines were subjected to cortical differentiation to generate the cell type affected in FAD. ICC was conducted on all control and FAD cell lines to investigate forebrain patterning at Day 25 via Forkhead Box G1 (FOXG1) and Paired box protein Pax-6 (PAX6) staining (Fig. 2A), as well as a neuronal phenotype at Day 100 with neuron-specific TUJ1 and lower cortical layer neuron marker TBR1 (Fig. 2B),

RNA from cell lines underwent qPCR analysis to assess the phenotype. Expression was normalized to housekeeping gene *RPL18a* prior to comparison. Expression of TBR1 (Fig. 2C, lower layer cortical neurons) was compared across cell lines, relative to the CTL2 line. There was a significant difference across cell lines (*P* = 0.02, one-way ANOVA) with a significant difference between the control and E280G.B lines (*P* = 0.02, Tukey’s multiple comparison test). There was no significant difference in SATB2 expression (Fig. 2D, upper layer neurons) or in TUJ1 (Fig. 2E, pan-neuronal marker) expression.

### Mutation-specific effects on Aβ species generation

**Figure 3:**
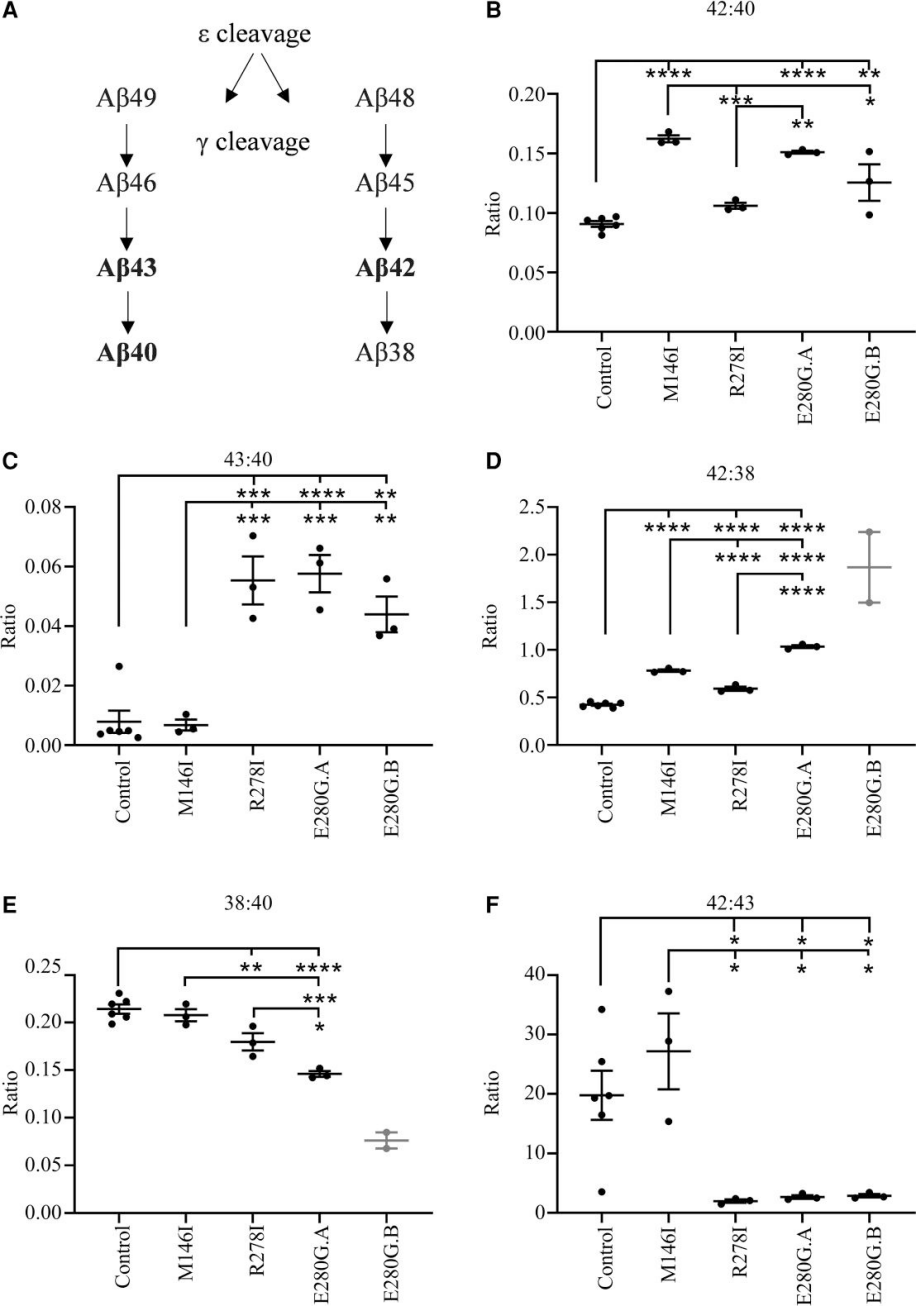
**Characterization of the Aβ profile of iPSC-derived neurons.** (**A**) Aβ cleavage down the Aβ48 or Aβ49 pathways. (**B**) Aβ42:40 used as a FAD biomarker. (**C**) Aβ43:40 used as a FAD biomarker. (**D**) Aβ42:38 as a measure of processivity. (**E**) Aβ38:40 a comparison of carboxypeptidase activity in the two cleavage pathways. (**F**) Aβ42:43 representing endopeptidase activity and tripeptide cleavage path choice. Each data point represents the mean value of duplicate technical repeats from a single induction. Control lines *n* = 6 (mean from 3 individual inductions from both control 1 + 2), *PSEN1* mutation lines *n* = 3 (mean from three individual inductions), except for E280G.B Aβ38 data points, where *n* = 2 as Aβ38 levels were below detection limits on the V-PLEX Aβ Peptide Panel 1 (6E10) Kit for 1 and thus have been excluded from comparisons involving Aβ38; however data points are still displayed for observation. Error bars represent mean and standard error of the mean (SEM), one-way ANOVA with Tukey’s test, *P < 0.05, **P < 0.01, ***P < 0.001, ****P < 0.0001

To explore Aβ processing down the Aβ48/49 pathways *in vitro* (Fig. 3A), we assessed Aβ peptide production in the control lines (pooled data), and the four FAD patient-derived lines (*PSEN1* M146I, *PSEN1* R278I and two *PSEN1* E280G). There was a significant difference in the Aβ42:40 ratio, indicative of both γ-secretase epsilon cleavage efficiency and selection of the Aβ48 or 49 pathway, across the cell lines (*P* < 0.0001). Compared with controls, there was a significantly increased ratio in the M146I, E280G.A and E280G.B lines (*P* < 0.0001, *P* < 0.0001, *P* = 0.006). M146I and E280G.A also had significantly increased Aβ42:40 ratios compared with R278I (*P* = 0.0003, *P* = 0.003). The M146I line also had a significantly increased Aβ42:40 ratio compared with E280G.B (*P* = 0.01), Fig. 3B.

The Aβ43:40 ratio, indicative of γ-secretase carboxypeptidase cleavage efficiency in the Aβ49 pathway, was significantly different between cell lines (*P* < 0.0001). Compared with controls, there was a significantly higher Aβ43:40 ratio in the R278I, E280G.A and E280G.B lines (*P* = 0.0001, *P* < 0.0001, *P* = 0.002). The Aβ43:40 ratio in the R278I, E280G.A and E280G.B lines was also significantly higher compared with M146I (*P* = 0.0004, *P* = 0.0002, *P* = 0.004), Fig. 3C.

The ratio of Aβ42:38, indicative of γ-secretase carboxypeptidase cleavage efficiency in the Aβ48 pathway, was compared across all lines, excluding E280G.B due to low *n* number (*n* = 2). The ratio was significantly different across the cell lines (*P* < 0.0001, ANOVA). Compared with controls, M146I, R278I and E280G.A had a significantly increased Aβ42:38 ratio (both *P* < 0.0001, Tukey’s test). The M146I line had a significantly increased ratio compared with R278I, and significantly reduced ratio compared with E280G.A (Both *P* < 0.0001). The R278I line had a significantly reduced ratio compared with E280G.A (*P* < 0.0001), Fig. 3D.

There was a significant difference across the lines (excluding E280G.B) in the ratio of Aβ38:40 (*P* < 0.0001), which indicates γ-secretase epsilon cleavage efficiency in the Aβ48:49 pathways. Controls had a significantly increased Aβ38:40 ratio compared with R278I and E280G.A lines (*P* = 0.008, *P* < 0.0001). The M146I line had a significantly increased Aβ38:40 ratio compared with E280G.A (*P* = 0.0002). R278I also had a significantly increased Aβ38:40 ratio compared with E280G.A (*P* = 0.02), Fig. 3E.

Pathway 43, also indicative of γ-secretase epsilon cleavage to the Aβ48 or 49 pathway, significantly differed across cell lines (*P* = 0.002). Compared with controls, there was a significantly decreased Aβ42:43 ratio in the R278I, E280G.A and E280G.B lines (*P* = 0.04, *P* = 0.05, *P* = 0.05). Additionally, compared with M146I, there was a significantly decreased Aβ42:43 ratio in the R278I, E280G.A and E280G.B lines (*P* = 0.01, *P* = 0.01, *P* = 0.01), Fig. 3F.

These data support *PSEN1* mutation-specific effects on APP processing and Aβ species generation. Specifically, the data from E280G support a relative increase in the generation of Aβ42 as well as a relative increase in Aβ43, explained by reduced processivity in both tripeptide cleavage pathways (48 > and 49>).

### PSEN1 autoendoproteolysis is affected by the E280G mutation

**Figure 4:**
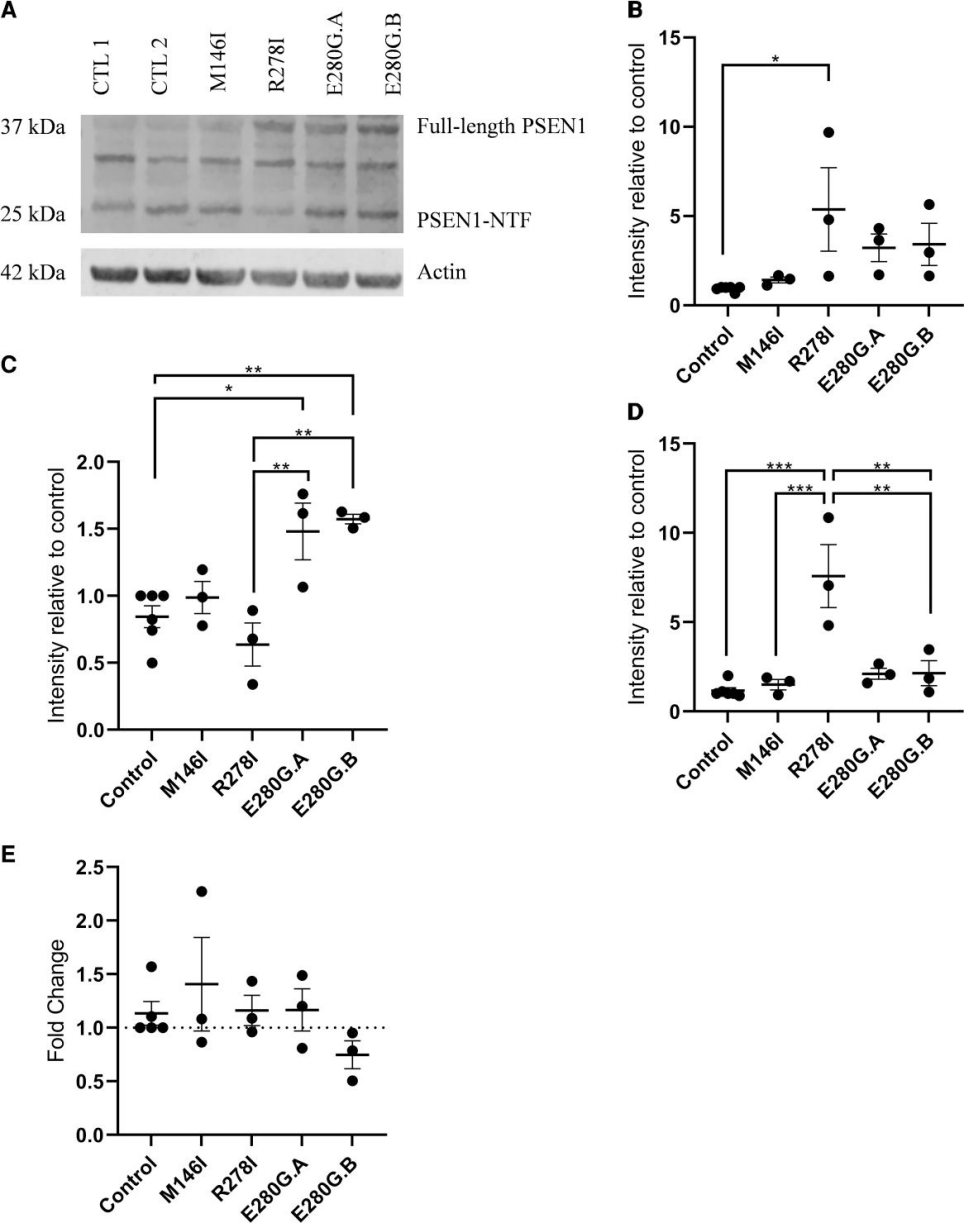
**PSEN1 peptide autoproteolysis and maturity in neurons.** (**A**) Representative western blot suggesting increased full-length PSEN1 in R278I and E280G lines and decreased PSEN1-NTF in the R278I line. An unidentified unspecific band is present at 33–34 kDa. (**B**) Full-length PSEN1 was significantly higher in the R278I line compared with control. (**C**) PSEN1-NTF was significantly higher in the E280G lines compared with the control, M146I and R278I lines. (**D**) Full-length PSEN1 relative to PSEN1-NTF was significantly higher in the R278I line compared with control, M146I and E280G lines. Each datapoint (4B–D) represents relative band intensity (normalized to CTL2) from a single induction. (**E**) No significant difference in *PSEN1* expression across cell lines, each datapoint represents normalized expression from a single induction, run in triplicate. One-way ANOVA with Tukey’s multiple comparison, error bars represent mean and SEM, *P < 0.05, **P < 0.01. Uncropped western blots for showing PSEN1 and Actin for each induction are provided in Supplementary Fig. 2

*PSEN1* mutations have been shown to alter γ-secretase stability.^38,41^ By using western blots to probe for the PSEN1-NTF, the presence of cleaved and full-length PSEN1 was assessed in the neurons, Fig. 4A.

Firstly, the amount of full-length PSEN1 (46 kDa), relative to actin, was normalized to CTL2. Protein expression was then compared between the control lines (pooled data), and the four FAD patient-derived lines (*PSEN1* M146I, *PSEN1* R278I and two *PSEN1* E280G). Full-length PSEN1 levels statistically differed across lines (*P* = 0.04), with significantly increased levels in the R278I line compared with the control group (*P* = 0.03), with a notable trend for increased levels in the E280G lines, see Fig. 4B.

Secondly, the amount of PSEN1-NTF (25 kDa), relative to actin and normalized to CTL2, was then compared between the control lines (pooled data), and the four FAD patient-derived lines. PSEN1-NTF levels indicate effective autocleavage of PSEN1 into the N and C-terminal fragments. PSEN1-NTF significantly differed across lines (*P* = 0.0007). Compared with the control group, PSEN1-NTF was significantly higher in the E280G.A and E280G.B lines (*P* = 0.02 and *P* = 0.006, respectively) and significantly higher in the E280G.A and E280G.B lines compared with the R278I lines (*P* = 0.005 and *P* = 0.002, respectively), see Fig. 4C.

Finally, the levels of full-length PSEN1 relative to PSEN1-NTF, normalized to the CTL2 line was compared between the control lines (pooled data), and the four FAD patient-derived lines. This ratio was significantly different across lines (*P* < 0.0002) with higher full-length PSEN1 levels in the R278I line compared with the control group (*P* < 0.0001), M146I line (*P* = 0.0008), E280G.A line (*P* = 0.002) and E280G.B line (*P* = 0.002), see Fig. 4D.

qPCR data revealed no significant difference in the expression levels of PSEN1 across the control lines (pooled data), and the four FAD patient-derived lines (Fig. 4E). Together, these findings suggest that the *PSEN1* E280G results in an accumulation of full-length PSEN1, *i.e.* the form that exists prior to autoproteolysis and maturation of PSEN1 protein fragments. However, unlike the R278I mutation that shows concurrently reduced levels of PSEN1-NTF, the data suggest no such effect of the E280G mutation.

### Aβ peptide deposition in human post-mortem brain tissue

Post-mortem cortical brain tissue from three individuals with FAD carrying the same *PSEN1* mutations that were studied in our cell lines (M146I, R278I and E280G) were available for immunohistochemical investigation. Note, the tissue and iPSC lines are not from the same individuals. The percentage area of Aβ-specific antibody coverage was assessed in the temporal and occipital cortices.

**Figure 5:**
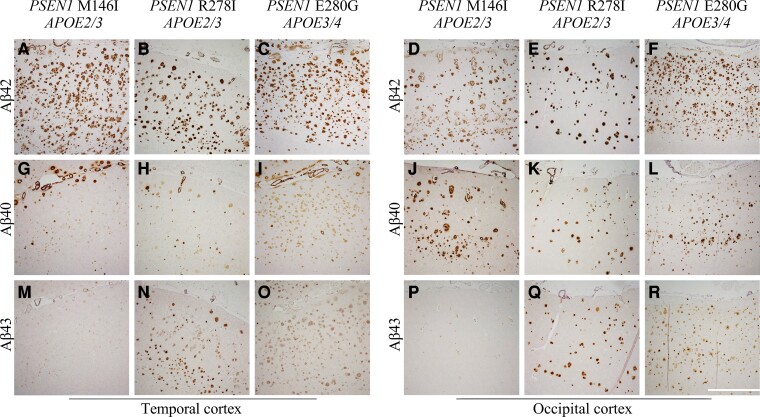
**Cortical Aβ peptide deposition.** Representative images of IHC performed on temporal cortex using antibodies specific to Aβ42 (**A–F**), Aβ40 (**G–L**) and Aβ43 (**M-R**). White scale bar representing 100 µm applies to all images

Immunohistochemical analysis displayed intense Aβ42 deposition in all cases in the temporal (Fig. 5A–C) and occipital cortices (Fig. 5D and F). Aβ42 was lowest in comparative regions in the R278I case (8.85 and 3.69%) compared with M146I and E280G mutations. In the temporal cortex the M146I and E280G cases had similar coverage (16.20 and 15.15%, respectively) while the MI146I had lower coverage in the Occipital cortex (6.53%) compared to the E280G case (12.70%).

Aβ40 pathology was present in all cases, notably as CAA, and cortical deposition (Fig. 5G–L). Aβ40 was lowest in the R278I case (2.01 and 2.52%). In the temporal cortex, the E280G case had greater coverage (7.89%) compared to the M146I case (3.12%), however, this was reversed in the occipital cortex with greater coverage in the M146I case (6.04%) compared with the E280G case (3.86%).

Aβ43 IHC shows mutation-specific differences in Aβ43 deposition (Fig. 5G–R). Aβ43 deposits can be observed as CAA and the prominent core of cored plaques, with varying intensity of cortical deposition and plaque type within and between cases. The lowest Aβ43 was observed in the M146I case (0.09 and 0.14%), with greater coverage in the R278I (4.62 and 3.70%) and E280G cases (2.92 and 4.91%). These findings mirror the results for high Aβ43 levels in conditioned media in the R278I and E280G cell lines.

## Discussion

The pathogenic effect of mutations in PSEN1 is mediated through alterations in APP processing with different mutations producing distinct effects on that processing. This may potentially underlie some of the clinical heterogeneity seen in FAD mutation carriers. Here, for the first time, we have defined the consequences of the *PSEN1* E280G mutation, which is associated with atypical clinical (spastic paraparesis) and pathological features (amyloid plaques of the cotton wool type). We have shown that in patient-derived iPSC-cortical neurons, the E280G mutation leads to increased ratios of Aβ42 and of Aβ43 compared with other length peptides, which is linked with both reduced PSEN1 autoendoproteolysis and relative abundance of both full-length and PSEN1-NTF.

One way in which mutations can affect PSEN1 is via the initial endopeptidase activity of γ-secretase.^42,43^ Mutations which effect the endopeptidase activity could affect both absolute Aβ levels, and which pathway of Aβ production is selected, leading to altered ratios of the peptides within those pathways.^22^ Whilst *PSEN1* mutations vary in whether or not they alter the endopeptidase activity of γ-secretase, they have been found to consistently decrease the efficiency of its subsequent carboxypeptidase activity by destabilizing enzyme–substrate complexes between γ-secretase and the Aβ peptides it is processing.^44^ The destabilization and compromised carboxypeptidase activity results in the release of longer Aβ peptides which are more prone to aggregation^6,15,22^ This in turn leads to more oligomeric Aβ and greater extracellular deposition.^23^ We observed a greater Aβ42:38 ratio in the M146I and E280G.A lines, compared with controls, indicative of consistently impaired carboxypeptidase activity in the Aβ48 pathway for these lines. Mutations near the autoendoproteolysis site are proposed to inhibit the cleavage that converts the full-length form into the mature, cleaved product.^26^ This can then lead to altered processivity by the enzyme.^27^ Interestingly, even within the full-length PSEN1, the catalytic pore is still able to be formed,^28^ which suggests that γ-secretase activity can still occur, although it is likely reduced, possibly explaining altered Aβ peptide production in mutations near this location. Such mutations in this region include PSEN1 R278I and E280G. Compared with controls and with M146I, the R278I and E280G lines displayed an increased Aβ43:40 ratio, indicating reduced carboxypeptidase activity in the Aβ49 pathway in a mutation dependant manner. Mutations not within the catalytic domain can likely affect the protein structure/stability and this may also impact interactions with substrates which will contribute to disease.^20,24,41,44^ For instance, the M146I mutation is not within the catalytic domains, yet displayed altered Aβ production profiles compared with controls, and other mutations.

Western blot analysis revealed a significantly increased full-length PSEN1 in the *PSEN1* R278I cell line compared with the control group. This has previously been shown for the R278I line, with a significant increase in full-length PSEN1 observed, and has been linked to Aβ43 production.^17,19,29,30^ It has also been observed for the E280G mutation,^20^ with our results suggestive of a trend for increased full-length PSEN1 in the E280G cell lines, although this did not reach significance.

The levels of PSEN1-NTF protein were also observed to be higher, and significantly so in the E280G neurons. As PSEN1 expression was not higher in these lines, it suggests that in the E280G lines there is an accumulation of PSEN1-NTF. This increase in PSEN1-NTF may explain the high levels of Aβ42 observed in these lines, in addition to the high Aβ43.

Finally, the level of full-length relative to PSEN1-NTF was significantly higher in the R278I line, which reflects the increase in products of the Aβ49 pathway only in this line. This measure also highlights that the R278I mutation specifically affects the processing of full-length to cleaved PSEN1, compared with other lines.

Similar to reported findings for the R278I mutation, the data presented in this study supports a similar effect of the E280G mutation, whereby PSEN1 protein maturation is affected. This is closely linked to the relative increase in Aβ43 production. The findings by western blot corroborate with the results from the ELISA analysis, where high levels of Aβ43 were observed in the *PSEN1* R278I and E280G mutation lines. It is likely that the mutation location hinders the processivity of full-length to cleaved PSEN1. The R278I and E280G mutations have a similar impact on Aβ ratios, however, the differential impacts on protein maturity effect may impact the amounts of mutant PSEN1 that are incorporated into the γ-secretase complex. Thus, absolute levels of Aβ could differ. To investigate this, isogenic lines with the homozygous mutations can be generated, as has previously been done for the PSEN1 Intron 4 deletion mutation.^45^This would allow the impact of these mutations to be assessed on protein maturity, Aβ ratios, and absolute Aβ levels, in the absence of a WT PSEN1 allele. It should be noted there was some variability between the two E280G lines. We cannot rule out that this could be due to the karyotypic abnormality in the E280G.A line in the 17q region. Although we were not able to identify the exact region that is duplicated, it is worth noting that the *MAPT* gene is located on chromosome 17q, encoding for tau, which forms pathological deposits in multiple dementias including FAD. Reassuringly, analysis of gene expression profiles in this line was normal (Supplementary Fig. 1) suggesting a minimal effect of this abnormality on stem cell health.

The mutation-associated differences in PSEN1 and subsequent Aβ production identified in this study are of particular interest in view of the distinct clinical and pathological features associated with the mutations we studied. E280G and R278I mutations both lie within exon 8, near the endoproteolytic cleavage domain. Mutations that are post-codon 200, and particularly within exon 8, have been linked to clinically atypical cognitive presentations and associated with spastic paraparesis.^32^ Motor symptoms have also been shown to be more frequent in *PSEN1* post-codon 200 cases.^46^ Additionally, there are observed differences in age at onset related to *PSEN1* mutation location, with mutations beyond Codon 200 typically associated with a slightly later age at onset.^32,47–49^ Mutations beyond Codon 200 have also been found to show histological differences, with more severe amyloid deposition in the vasculature as CAA compared with mutations before Codon 200.^49^ The study by Mann *et al.*^49^ also found that the amount of cortical deposition of Aβ42 correlated with cell line production of Aβ42 in matched mutation cell line/tissue comparisons. While our case numbers were small, our histological observations support mutation-associated Aβ histological profiles and the Aβ specific IHC in matched mutation human cortical tissue corroborated our immunoassay findings. Both *PSEN1* R278I and E280G mutation cases displayed greater Aβ43 deposition, while M146I did not, supporting mutation-specific alterations in the Aβ49 pathway. Interestingly, a recent report demonstrated that the *PSEN1* L435F mutation also leads to increased Aβ43 in iPSC and post-mortem tissue, and thus our work expands the range of mutations known to specifically impact this peptide.^50^ Aβ40 deposition was apparent in all cases, which may be unexpected in the M146I case, as the immunoassays indicate alterations primarily in the Aβ48 pathway. However, Aβ40 is the most abundant Aβ peptide observed in human CSF including in Alzheimer’s disease patients,^51,52^ and in human cell lines^53,54^ so some deposition could well be expected. Additionally, this case displayed Aβ40 primarily as CAA, a predominant component of CAA.^49,55,56^ The M146I case has an apolipoprotein (*APOE*) ε2/ε3 genotype. Generally, the *APOE* ε4 allele is linked to sporadic CAA, with a meta-analysis indicating *APOE* ε4 as a risk variant in grouped dementia patients and controls while *APOE* ε2 may result in less CAA,^57,58^ although a positive *APOE* ε4 carrier status and CAA severity were not associated in a study of *PSEN1* mutation cases.^59^ Post-mortem analysis in 93 sAD patients revealed a significant association of CAA pathology with the *APOE* ε4 allele, with increasing *APOE* ε4 allele number associated with increasing severity of CAA.^60^ However, the role of the *APOE* genotype in CAA is not clear cut, as carriers of the *APOE* ε2 allele have been found to have more severe CAA than those with the *APOE* ε3/ε3 genotype, despite the *APOE* ε2 allele conferring lower risk for Alzheimer’s disease.^61^ Additionally, Alzheimer’s disease patients who were *APOE*2 carriers had worse small vessel disease ^62^ and a meta-analysis found *APOE* ε2 to be associated with certain markers of cerebrovascular disease.^63^ These factors may contribute to the association between *APOE* ε2 and CAA. Thus, the *APOE* ε2 carriership in the M16I mutation case may influence the Aβ40 pathology displayed in this case. However, the R278I case also has an *APOE* ε2/ε3 genotype, which could also impact the histological observations. In future work, it would be of interest to investigate the Aβ peptides deposited in a larger number of individuals carrying the same FAD mutation but different *APOE* genotypes. Such larger studies will be needed in order to fully investigate correlations between clinical and pathological features and mutation-associated differences in Aβ processing. While studying pathology and iPSCs from the same individuals would be a gold standard, the altered Aβ ratios observed in iPSC studies are conserved in FAD and also in sAD human tissue, validating the use of iPSCs as models and implying γ-secretase alterations do occur *in vivo* and can occur in sAD.^64^ Furthermore, the approach used here has enabled some confirmation of cell line results to human pathological tissue, highlighting the validity of the models to recapitulate FAD mechanisms and their appropriateness to investigate clinical and pathological associations.

## Supplementary Material

fcac321_Supplementary_Data

## Data Availability

The authors confirm that all data supporting the findings of this study are available within the article and readily available upon request. Uncropped western blots for the data provided in Fig. 4 are provided in Supplementary Fig. 2.

## Abbreviations

Aβ =: amyloid beta
APP =: amyloid precursor protein
CAA =: cerebral amyloid angiopathy
CWP =: cotton wool plaque
DAP = 4′ =: ,6-diamidino-2-phenylindole
FAD =: familial Alzheimer’s disease
ICC =: immunocytochemistry
IHC =: immunohistochemistry
iPSCs =: induced pluripotent stem cells
NTF =: N-terminal fragment
PSEN1 =: Presenilin 1
RIPA =: radio immunoprecipitation assay buffer
SNP =: single nucleotide polymorphism
WT =: wild type
