# Supplementary material for: The PSEN1 E280G mutation leads to increased amyloid-β43 production in induced pluripotent stem cell neurons and deposition in brain tissue
Source: Brain Commun. 2022 Dec 7;5(1):fcac321. doi: 10.1093/braincomms/fcac321 (PMC9847549; doi:10.1093/braincomms/fcac321)
Supplement: fcac321_Supplementary_Data [file fcac321_supplementary_data.pdf]

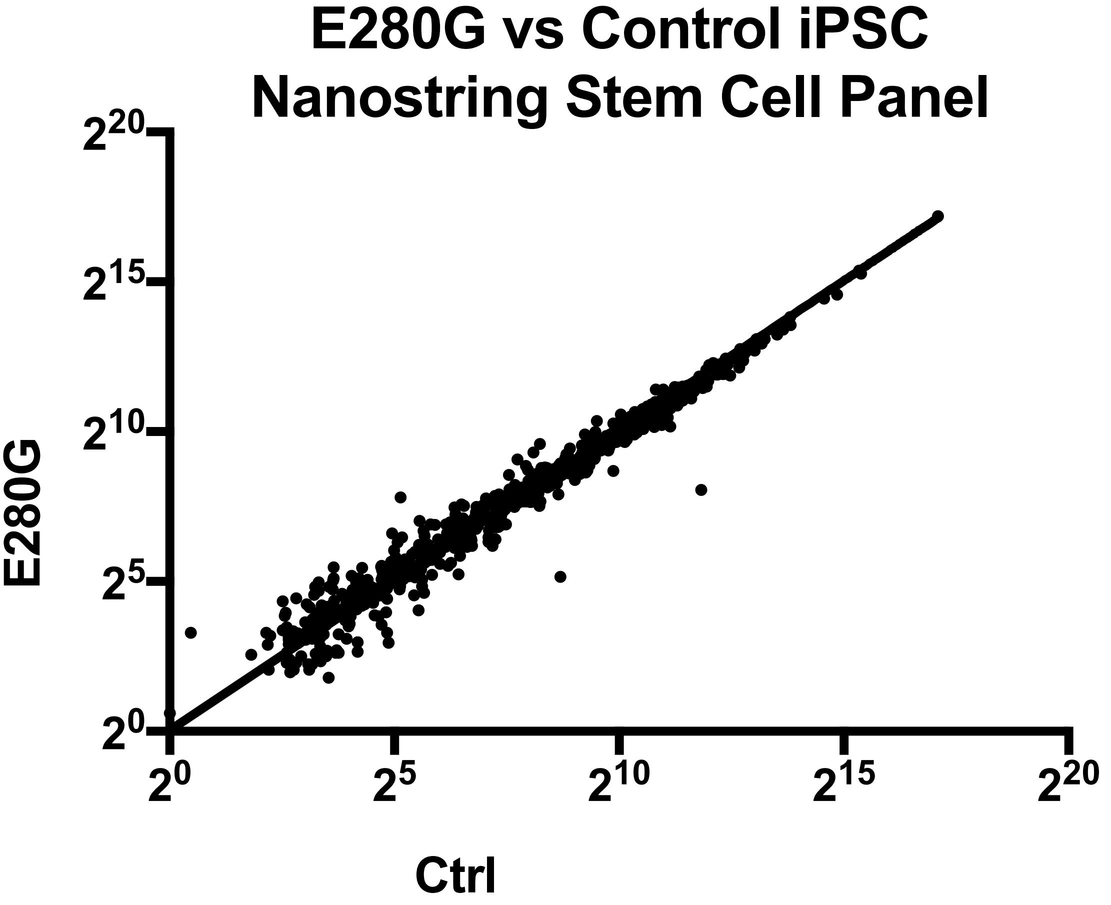

Supplementary Figure 1: *Analysis of grouped data to compare control lines (CTL1 and CTL2) with E280G lines (E280G.A and E280G.B), R^2 = 0.9932, p*<0.0001, Linear regression.

Induction 1

Presenilin1

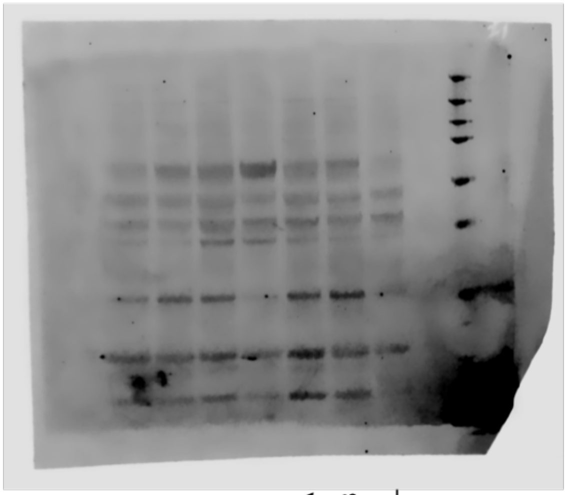

Actin

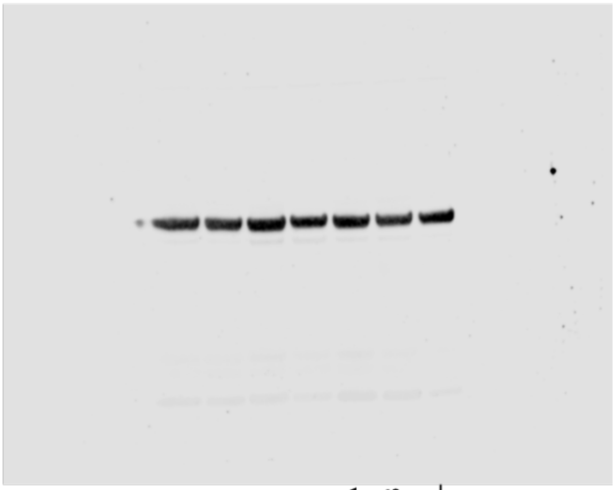

Induction 2

Presenilin1

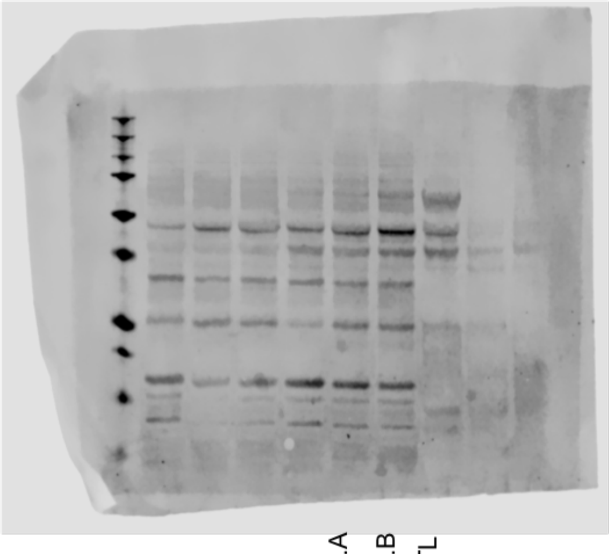

Actin

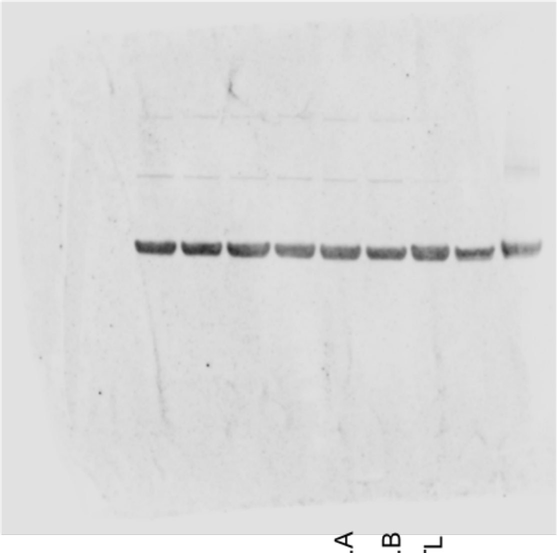

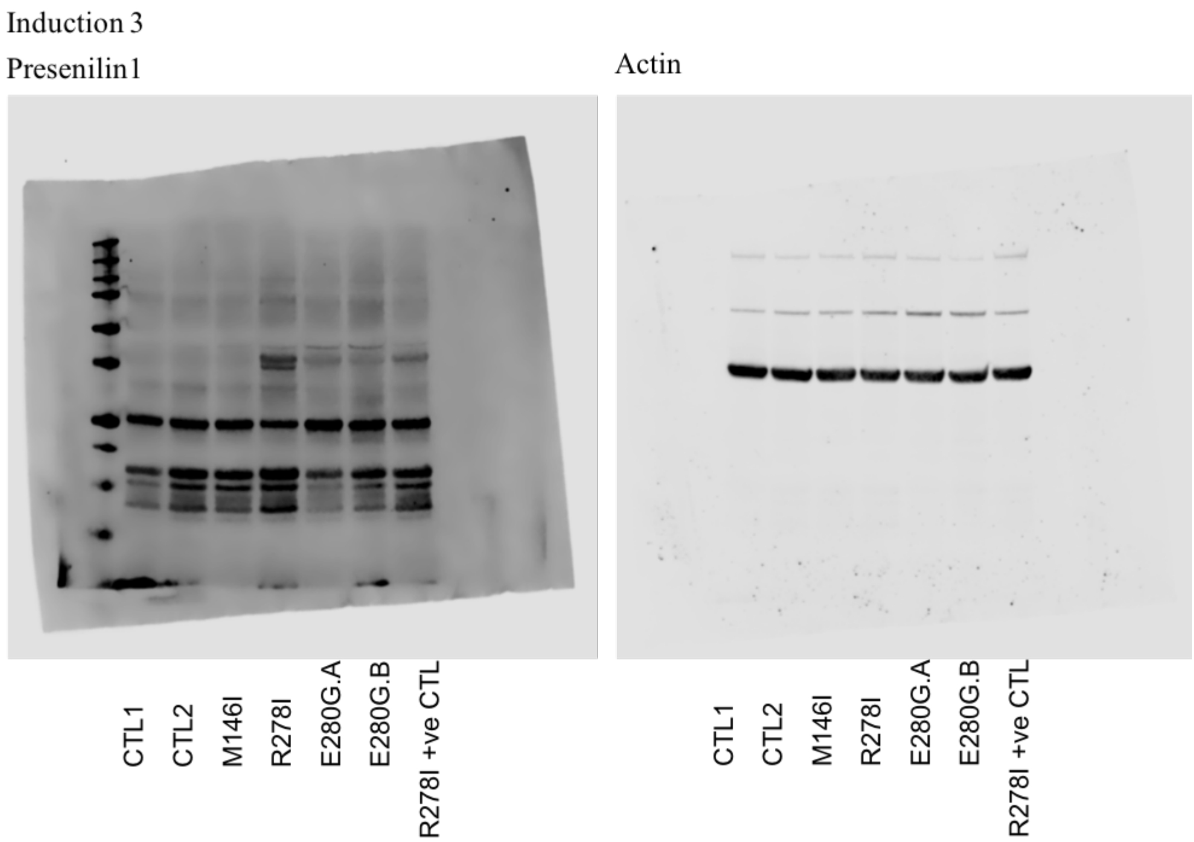

Supplementary Figure 2: uncropped western blot images from each induction, probed for PSEN1 and Actin.
